# Early Outbreak of 2009 Influenza A (H1N1) in Mexico Prior to Identification of pH1N1 Virus

**DOI:** 10.1371/journal.pone.0023853

**Published:** 2011-08-31

**Authors:** Ying-Hen Hsieh, Stefan Ma, Jorge X. Velasco Hernandez, Vernon J. Lee, Wei Yen Lim

**Affiliations:** 1 Department of Public Health, China Medical University, Taichung, Taiwan; 2 Center for Infectious Disease Education and Research, China Medical University, Taichung, Taiwan; 3 Epidemiology and Disease Control Division, Ministry of Health of Singapore, Singapore, Singapore; 4 Programa de Matemáticas Aplicadas y Computación, Instituto Mexicano del Petróleo, México City DF, Mexico; 5 Departamento de Biociencias e Ingeniería, Interdisciplinary Center for Research and Studies on Environment and Development-National Polytechnic Institute, México City DF, Mexico; 6 Headquarters Medical Corps, Singapore Armed Forces, Singapore, Singapore; 7 Department of Epidemiology and Public Health, Yong Loo Lin School of Medicine, National University of Singapore, Singapore, Singapore; Center for Complex Networks and Systems Research, Indiana University at Bloomington, United States of America

## Abstract

**Background:**

In the aftermath of the global spread of 2009 influenza A (pH1N1) virus, still very little is known of the early stages of the outbreak in Mexico during the early months of the year, before the virus was identified.

**Methodology/Main Findings:**

We fit a simple mathematical model, the Richards model, to the number of excess laboratory-confirmed influenza cases in Mexico and Mexico City during the first 15 weeks in 2009 over the average influenza case number of the previous five baseline years of 2004-2008 during the same period to ascertain the turning point (or the peak incidence) of a wave of early influenza infections, and to estimate the transmissibility of the virus during these early months in terms of its basic reproduction number. The results indicate that there may have been an early epidemic in Mexico City as well as in all of Mexico during February/March. Based on excess influenza cases, the estimated basic reproduction number R_0_ for the early outbreak was 1.59 (0.55 to 2.62) for Mexico City during weeks 5–9, and 1.25 (0.76, 1.74) for all of Mexico during weeks 5–14.

**Conclusions:**

We established the existence of an early epidemic in Mexico City and in all of Mexico during February/March utilizing the routine influenza surveillance data, although the location of seeding is unknown. Moreover, estimates of R_0_ as well as the time of peak incidence (the turning point) for Mexico City and all of Mexico indicate that the early epidemic in Mexico City in February/March had been more transmissible (larger R_0_) and peaked earlier than the rest of the country. Our conclusion lends support to the possibility that the virus could have already spread to other continents prior to the identification of the virus and the reporting of lab-confirmed pH1N1 cases in North America in April.

## Introduction

It was reported that cases of the novel influenza A (H1N1) had begun to emerge in March of 2009 in Mexico, and by the first 2 weeks of April were beginning to be identified in Mexico and California [1]–[2]. This outbreak of influenza-like illness cases led to the first report made by the Mexican government to the Pan American Health Organization (PAHO) on 12 April. By the end of April, the epidemic had spread nation-wide with most of the cases being reported in Mexico City [2]. Epidemiological investigations of the early La Gloria outbreak led to an estimated date of first case on February 15 [3]. The same authors also estimated a time of the most recent common ancestor (TMRCA) of January 12, 2009 [95% Credible Interval (CrI): 3/11/2008 to 2/3/2009]. The novel H1N1 virus eventually swept across the globe, prompting the World Health Organization (WHO) to announce a pandemic of H1N1 virus (pH1N1) in June [4].

Most of the early suspect cases were hospitalized cases with severe acute respiratory disease [5], as early detection of outbreak through severe cases and notifications of clusters was typical. However, it is likely (especially in the case of influenza) that a larger number of cases that were not labeled as suspect or confirmed had occurred prior to the reporting of clinical cases, making it extremely difficult to determine the early transmissibility of the virus, via the basic reproduction number R_0_ or the number of secondary cases caused by an index case in an immunologically naïve environment, solely from the confirmed and hospitalized cases. By making use of the standard influenza surveillance data of Mexico, our aims are: (i) to establish the introduction of the virus in humans via the ascertainment of an early epidemic in February/March by utilizing only the regular government surveillance data; (ii) to estimate and compare its early transmissibility (R_0_) as well as the time of peak incidence (turning point) in Mexico City and all of Mexico at the early stages of the epidemic.

## Materials and Methods

### Data

For this study, we obtained the laboratory confirmed influenza case data of Mexico City (also known as the Federal District) and of all of Mexico for the years 2004–2009 from the Mexican Department of Health website [6]. In Mexico, influenza cases must be notified within 24 hours of confirmed diagnosis. Weekly confirmed influenza cases that were notified were reported by week of notification, and the data was available for the first 15 epidemiological weeks (or weeks) of each of these two years, before the reporting protocol regarding influenza cases changed due to the identification of pH1N1 virus in late April. (Here a week runs from Sunday to Saturday and must consist of at least 4 days, hence week 1 of 2009 starts on January 4.) In order to focus on the excess influenza cases that might have occurred in 2009 in Mexico during the first 15 weeks before the novel strain was identified, we compare the 2009 weekly influenza case data with the averages of the corresponding weekly data of 5 “baseline” years prior to 2009, i.e., 2004–2008.

**Figure 1:**
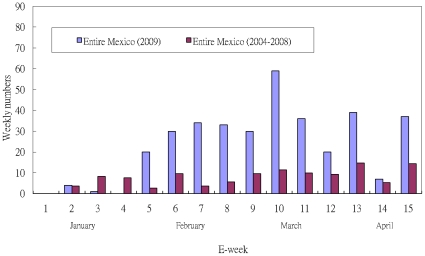
Epidemic curves of weekly reported cases of seasonal flu in Mexico City during the first 15 weeks of 2004-2008 and 2009 in (a) Mexico City (b) all of Mexico.

Data for this analysis was obtained from the weekly epidemiologic bulletin released by the Centro Nacional de Vigilancia Epidemiologica y Control de Enfermedades, Mexico. As part of a national programme of epidemiologic surveillance, the centre maintains surveillance over some 98 diseases considered of public health importance. All units within the health system (of which there are about 189 000 currently, distributed throughout the country) report through an electronic reporting system, on a weekly basis, the number of new cases of each of the disease under surveillance. This data is sent to the national centre where it is consolidated and analyzed. A weekly bulletin of new cases is then produced. Both acute respiratory infections and influenza are reported under this system [7]. In addition, Mexico has an influenza surveillance system, where monitoring centers which are disease control centers or hospitals designated by each state autonomously (Mexico has a federal system similar to the US) in all 32 states of Mexico have been established. These monitoring centers identify probable cases of influenza based on defined clinical criteria of patients attending their centers. Biological samples are obtained from these patients for laboratory confirmation. Confirmed cases are included in the weekly epidemiologic bulletin [8]. The data are given in Figure 1.

**Figure 2:**
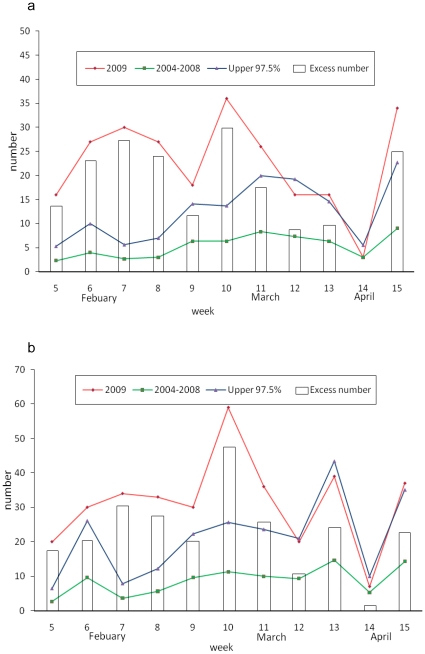
Excess weekly influenza case number in 2009 over the average weekly influenza case number of 2004-2008 in (a) Mexico City, weeks 5–9, 2009 (b) all of Mexico, weeks 5-12, 2009. Red line is the 2009 weekly influenza case data; Green line is the averaged 2004–2008 weekly influenza case data; blue line is the upper 97.5% levels; and the boxes are the weekly excess case numbers.

We compute the weekly number of excess influenza cases in 2009 over the average of the corresponding weekly case number in the “baseline years” of 2004–2008 in Mexico during the first 15 weeks; since most of these excess influenza cases that occurred in 2009 were likely caused by the novel pH1N1 virus strain. Excess influenza cases were observed during weeks 5–15 in both Mexico City and all of Mexico. The excess 2009 influenza case numbers over the corresponding 2008 data are also computed (see Figure 2). Moreover, the excess cases during weeks 5–11 in 2009 was statistically significant for both Mexico City and all of Mexico, as the excess numbers during this period were greater than the baseline weekly averages of 2004–2008 by more than one standard deviation (SD).

The underlying assumption here is that, while the testing rate for influenza was most likely very low during the first 15 weeks of 2009 before the identification of the novel pH1N1 virus, it was at similarly low levels throughout the same periods in 2004–2008. Consequently, the significant number of excess cases found during this period in 2009 over the baseline levels of the same periods during the previous five years are most likely due to the novel pH1N1 virus and hence scientifically meaningful to be used to quantify the early stages of the epidemic before the virus was detected and identified by the surveillance system.

### Richards Model

Unlike the more commonly used Susceptible-Infective-Removal (SIR) compartmental model which is used to describe the transmission dynamics of an infectious disease, the Richards model considers only the cumulative infected population curve with saturation in growth as the outbreak progresses, which is possibly caused by factors such as depletion of susceptibles or implementation of control measures. Although data by reporting date is often scrambled by artificial factors such as health system alertness, public response, and government responsiveness, the Richards model is useful to capture the temporal variations of an outbreak, in particular the turning points (or peaks and valleys of the incidence curve) which are at times results of these artificial factors.

The basic premise of the Richards model is that the incidence curve of a single wave of infections consists of a single peak of high incidence, resulting in an S-shaped cumulative epidemic curve and a single turning point of the outbreak. The turning point, denoted by t_i_, is defined as the point in time at which the rate of accumulation changes from increasing to decreasing and can be easily pinpointed by locating the inflection point of the S-shaped cumulative case curve [9]–[11]. This quantity, although subject to stochastic (“random”) variations and difficult to pinpoint (see [12] for a related discussion), has obvious epidemiologic importance, indicating the beginning (i.e., moment of acceleration after deceleration) of saturation of the S-shape cumulative case curve. Moreover, it is also the time of peak incidence for this particular wave of cases.

Richards [13] had proposed the following model to study the growth of biological populations: 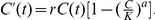 The prime “
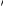
” denotes the time rate of change and the time unit is in weeks. *C(t)* is the cumulative number of cases at time *t* (in weeks) with t = 0 denoting the first week of the data, *K* is the cumulative case number over a single wave of outbreak, *r* is the per capita growth rate of the infected population, and *a* is the exponent of deviation. The explicit solution of the Richards model is 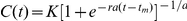. Here the parameter *t*_m_ is related to the turning point t_i_ of the epidemic (or the inflection point of the cumulative case curve) by the simple formula 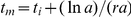, where *ln* denotes the natural logarithm function [9]–[11].

For the computation of the basic reproduction number R_0_, the formula 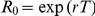 is used where T is the generation interval of the disease, the average time interval from onset of one infective to the time when the onset of his/her contacts occurs [11], [14]–[15]. It has been shown mathematically [16] that, given the growth rate *r*, the expression 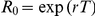 provides the upper bound of R_0_ regardless of the distribution of the generation interval that is being used. In recently years, the Richards model has been employed successfully to model infectious disease outbreaks including SARS [9]–[11], dengue [14]–[15], and the pH1N1 outbreak in the Southern Hemisphere [17] and in Canada [12].

## Results

**Figure 3:**
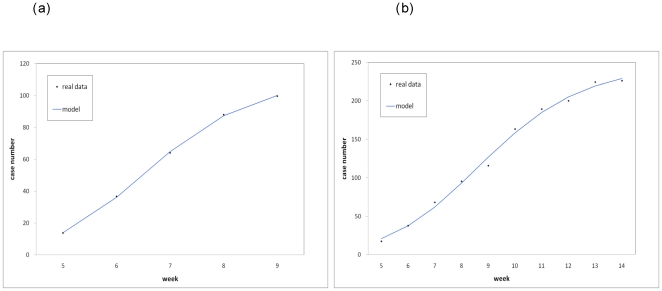
Model fit of the cumulative excess influenza case number in 2009 over the average weekly influenza case number of 2004-2008 in (a) Mexico City, weeks 5–9, 2009 (a) all of Mexico, weeks 5–14, 2009. Dots are the real data and the curves denote the theoretical case numbers estimated by the Richards model.

Fitting the Richards model to the excess influenza case number data, we obtained model fit for one-wave outbreaks during weeks 5–9 for Mexico City and during weeks 5–14 for all of Mexico. The results are given in Table 1 and Figure 3. Table 1 shows the estimation results for the turning point t_i_, growth rate r, cumulative case number K, and basic reproduction number R_0_ (assuming negligible pre-immunity). Since week 5 is t = 0 in our model, the result of t_i_ = 1.46 for Mexico City indicates that a turning point or a peak of the outbreak, where the case number started to level off, had occurred around mid-February during the 7^th^ week (February 15–21); while for all of Mexico with t_i_ = 3.56, the turning point occurred during week 9 (March 1–7).

**Table 1:** Estimation results using the weekly excess influenza case data in 2009 over the weekly average of 2004-2008 by reporting date during weeks 5–9 for Mexico City and during weeks 5-14 for all of Mexico.

Location(Time Period)	Turning point t_i_(95% C.I.)	Growth rate r(95% C.I.)	Cumulative case number K (95% CI)	R_0_(95% C.I.)
Mexico City(weeks 5-9)	1.46^1^(0*, 17.73)	1.69(0*, 12.94)	110(64, 160)	1.59(0.55, 2.62)
All of Mexico(weeks 5-14)	3.56^2^(0.0, 13.29)	0.81(0*, 2.31)	246(203, 289)	1.25(0.76, 1.74)

1 Denoting turning point during week 7 (February 15–21).

2 Denoting turning point during week 9 (March 1–7).

*max(0, lower bound).

Note that the cumulative case number is rounded off to the nearest integer. The actual cumulative excess number K for weeks 5–9 in Mexico City is 100 and for weeks 5–14 is 226 in all of Mexico. R_0_ was computed using the mean estimated generation interval of T = 1.91 days (95% CI: 1.30–2.71), which was estimated from early Mexico novel H1N1 data in La Gloria before April 30, 2009 [3].

For the purpose of computing R_0_, we make use of the mean estimated generation interval (and its 95% CI) of T = 1.91 days (95% CI: 1.30-2.71) as given in [3], which was estimated from early Mexico novel H1N1 data in La Gloria before April 30. The data fit to the explicit solution of the Richards model was performed using the software SAS with least-squares approximation tool.

The confidence intervals for the growth rate “r” were obtained empirically from the model fit to the data using SAS least squares estimation subroutine, where the estimation converges for data of weeks 5–9 (only 5 data points) for Mexico city and weeks 5–14 (10 data points) for all of Mexico. Subsequently, the confidence interval for Mexico City is very large in comparison to that of all of Mexico, reflecting the difference in data size. In contrast, the confidence intervals for the basic reproduction number R_0_ were computed via the formula given in the previous section using the mean estimates and variances of “r”, which was obtained empirically, and the same mean estimate and variance of the generation interval T [3]. As a result, the effect of different data size (5 data points vs. 10) was diluted through the computation.

## Discussion

The estimated basic reproduction number for the early outbreak based on the excess influenza cases was 1.59 (0.55 to 2.62) for Mexico City during weeks 5–9, and 1.25 (0.76, 1.74) for all of Mexico during weeks 5–14 which are comparable to most of the estimates (between 1.21–1.88) obtained by other modeling studies of the spring outbreak in Mexico [3], [18]–[22] and also in good agreement with estimates for the rest of the world (see, e.g., [12], [17], [23]–[28], or Table 2 of [21] for a list of estimates for various countries).

The difference in the estimates of R_0_ for Mexico City and for all of Mexico (which is comparatively smaller) may be indicative of the difference in levels of underreporting of influenza cases in different parts of Mexico before the outbreak became widely known, especially outside of Mexico City. Alternatively, the low transmissibility of virus in all of Mexico perhaps indicates that the outbreak might have not yet reached all parts of Mexico at this early stage. Therefore, the higher estimate of R_0_ using excess case number in Mexico City may be a more reliable estimate of the true transmissibility of pH1N1 virus. However, 95% confidence interval for Mexico City is wider, mainly due to its smaller data size.

The estimate obtained for the cumulative case number K of this early wave of epidemic, as given in Table 1, are obviously in good agreement with the observed excess case data, since no underreporting or other type of bias is considered in the model. Moreover, the model is a phenomenological model constructed to describe an observed phenomenon, i.e., the confirmed case number, which differs from the work of Lipsitch et al. [29] and Colizza et al. [30] that estimate the actual case number by taking into account of the possible bias in the surveillance methods. However, one could theoretically estimate a range of underreporting by dividing the estimated range of cumulative confirmed case number obtained in this work by the estimated case number obtained through the methods in [29]–[30].

The turning point or the peak time of the outbreak in Mexico City took place around week 7 (February 15–21) before the La Gloria outbreak. For the outbreak in all of Mexico the turning point occurred around week 9 (March 1–6), indicating that the outbreak in Mexico City had peaked two weeks earlier than the rest of the country. Our finding of an early outbreak by early February (starting on week 5 or February 1–7) corroborates the first reported case of the La Gloria outbreak occurring around February 15 through epidemiological investigations and is consistent with the estimated TMRCA reported by Fraser et al. [3]. Similar to the la Gloria data, our excess influenza case data, provide evidence that by February the virus may have already spread in Mexico City as well as Gloria, and perhaps other towns in the area that did not have the urban surveillance of Mexico City or the epidemiological study that was carried out in la Gloria. Subsequently, the virus could have been seeded in la Gloria or anywhere else in Mexico, but most likely before February 15.The earliest available confirmed case data on Mexico pH1N1 reported in [22] spans the time period of March 11–May 2 starting in the middle of week 10, after the peak for the early wave in Mexico City (week 7) and for all of Mexico (week 9) according to our study results. Therefore this dataset unfortunately cannot be used to ascertain whether there was a wave before March 11. Moreover, the limited excess case data prevents us from a thorough statistical investigation. For this reason the paucity of data at such early stage makes our analysis using the routine influenza surveillance data valuable, as it contributes to filling a void in the current understanding and knowledge regarding the early days of the 2009 pH1N1 epidemic.

A recent modeling study in Australia [31] suggests that community transmission of pH1N1 was well established in the state of Victoria in April when the virus was first identified in North America, which is compatible with modeling results that take into account of the international travel (e.g., [32]). Moreover, serologic evidence from a children-based household study in central Taiwan indicates that some serum samples taken from the 306 subjects had more than 4-fold increases in their hemagglutination inhibition (HI) titers against pH1N1 between October/2008-February/2009 and April of 2009 [33], suggesting that these individuals had already been infected with the 2009 pH1N1 virus before the first laboratory confirmed pH1N1 case in Taiwan arrived from North America on May 19 [34]. These studies suggest the likely earlier start of the international spread of the pH1N1 virus prior to April and before the novel strain was first identified, thus giving credence to an earlier time course for an early outbreak in Mexico by February.

**Figure 4:**
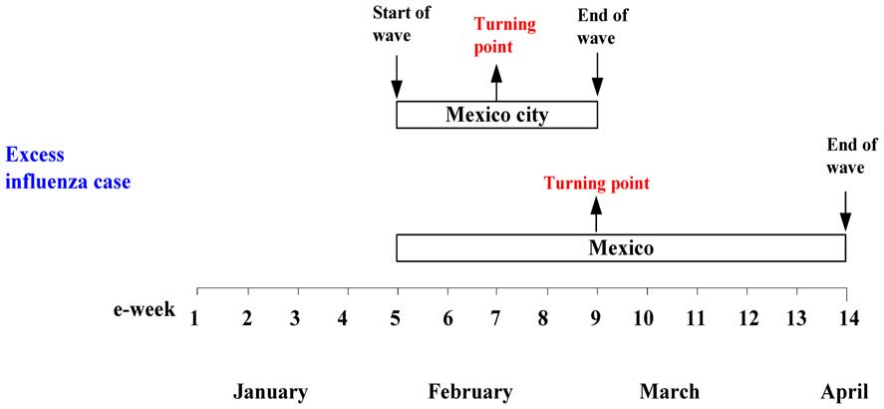
Chronological timelines of the early 2009 pH1N1 epidemic in Mexico.

The complete chronological timeline of the early outbreak in Mexico and Mexico City, as modeled by the Richards model, is graphically illustrated in Figure 4. We note that the beginning of substantial excess influenza cases in week 5 (February 1–7) coincides with the Día de la Candelaria, or the Candlemas long weekend holiday in Mexico ending on February 2, which was a Monday and the second day of week 5. It was the only holiday in February and the increased travel over this long weekend holiday might have contributed to this early outbreak in all of Mexico in February. It is also interesting to note that the turning point (or the time of peak incidence) of the outbreak obtained by using the influenza case data and excess influenza data are in exact agreement, at week 7 and week 9 respectively for Mexico City and for all of Mexico.

The Candelaria feast in the first week of February (or week 5), widely celebrated locally in relatively large towns and cities all over Mexico including the towns of Jalapa and Perote near la Gloria where local residents and visitors mix for two or three days, is very likely the reason we were able to see for the fist time any *indirect* evidence of the new strain in early February, via the excess influenza case data. However, while the increased travel led to increased spatial dissemination once the outbreak has started, it was unlikely the cause of epidemic, as the early phase is prone to stochastic fluctuations and high mixing would tend to favor the epidemic to start.

We note also that the increases in the last week of the data (week 15 or April 12–18) in Figure 1 is most likely an early indication of a new surge in cases which, along with the announcement by USCDC on April 23 regarding the identification of the novel swine influenza [35], prompted the authority to change the case reporting protocol in the following week (week 16). However, the early outbreak prior to the identification of pH1N1 is the main focus of our study. Since the reporting protocol regarding influenza cases was changed after week 15 due to the identification of pH1N1 virus in late April, we are unable to extend this modeling study into April and May for the well-known spring wave.The pH1N1 influenza virus is a new variant whose confirmed identity was established well into 2009. Its existence in Mexico prior to the mid-March is still a matter of debate [36]–[39]. The Health Department of the State of Veracruz, where the town of La Gloria is located, reported the existence of several hundred cases of “respiratory disease” in the area in late 2008 [40]. There are much discussion regarding how prevalent pH1N1 had been before March but no clear argument can be sustained due to the lack of reliable data. Strictly speaking, the argument reported in this work is sound but not conclusive for the same reason. Nevertheless, it is an interesting hypothesis that puts forward evidence that pH1N1 indeed was cocirculating with other influenza-like diseases (including seasonal influenza) at least as early as in February, which is corroborated by the sharp increase in the confirmed influenza case number in Mexcico starting in February (Figure 1 and [41]). We also note that co-infection of pH1N1 with seasonal H1N1 has also been reported in New Zealand during the Southern Hemesphere's winter influenza season [42].

Multiple waves are commonly seen in infectious disease outbreaks for a variety of reasons such as the occurrence of a large new cluster of infection (2003 SARS in Toronto [10]), climatological events affecting the vector population (2007 dengue in Taiwan [15]), or the implementation of containment policies (Mexico pH1N1 in April-June). For Mexico in February 2009, the short Candelaria feast weekend may well have increased contacts and therefore infections temporarily, but the disease in infected individuals was manifested only when the local population was back to below threshold (and all transients had already left). It follows that there were cases but not sustained epidemic outbreaks, and that the disease peaked and subsided quickly by March with only marginal transmission until April with the Easter Holy Week throughout Mexico and particularly in the surroundings of Veracruz.

In 2009, the Holy Week was April 5–12 (essentially week 14) with one more (the following) week to cover the whole 2-week vacation period in Mexico for all of the federal school system. In general, everybody is on vacation for the weekend starting the Holy Thursday and Holy Friday (on April 9 and 10, respectively). In short, no epidemic outbreak developed after the Candelaria feast in February with only few reported cases (or stochastic oscillations in incidence), until the sharp increase in cases in week 15 right after the long and extensive Holy Week holidays where the increased population mixing generated by tourists and visitors produced the conditions for an exponential increase in contacts and the subsequent surge in case.

In summary, we have established the existence of an early epidemic in Mexico City and all of Mexico during February/March utilizing only the routine influenza surveillance data. Moreover, we obtained estimates for R_0_ as well as the time of peak incidence (turning point) for Mexico City and all of Mexico, which indicate that the early epidemic in Mexico City in February/March had been more transmissible (larger R_0_).

Limitations of this study include the fact that data was obtained from open sources and hence is subject to underreporting. This dilemma is further complicated by the lack of knowledge regarding the asymptomatic and mild cases of influenza [3]. Our use of laboratory confirmed case data certainly suffers from underreporting issues. However, the confirmed cases constitute a sample of the clinical cases, albeit a non-random one. Assuming that there was a consistent testing rate throughout this time period when the novel strain was yet unidentified, the time series of excess confirmed case data would truthfully reflect the temporal trend of the early epidemic, and hence can be reasonably utilized to quantify the initial growth and the turning points that had occurred during this period via the Richards model. The main theme of this paper is to propose *some* evidence of an early wave, as indicated by our endeavor to fit the excess influenza case data in February and March. The duration of the model fit *for the early wave* ends on week 9 for Mexico City (Figure 3a) and week 14 for all of Mexico (Figure 3b), when the testing rates were still mostly comparable to testing rates during past winter seasons. We note, however, that the uncertainty (as measured by the 95% CIs) for the turning points is quite large. The problem obviously lies in the limited data size that is available. Hence although our results do shed lights on the existence and transmissibility of an early epidemic, the estimates for the turning points are less certain and should be viewed with caution.

Ideally, parameter estimation using the Richards model should start with the earliest possible data, since the initial growth rate of an epidemic would be an important part of the estimation. For Table 1, the estimates were obtained by assuming week 5, the first week of statistically significant number of excess influenza cases, to be the starting date (t = 0), which is reasonable given our assumption that the excess cases were mainly due to the novel pH1N1 virus.

It had been said that the only thing certain about influenza viruses is that nothing is certain [43]. Influenza and influenza-like illness surveillance numbers, although less sensitive and subject to stochastic errors, contain important information pertaining to infections and spread of influenza in the community that one can utilize to reflect the unconfirmed cases that are undoubtedly present in the community, and hence are extremely useful data in retrospectively ascertaining the early outbreak via its temporal changes and reproduction number. Moreover, by making use of the routine influenza surveillance data to determine whether the excess case data can be fitted to a single-wave outbreak, our work demonstrates the possibility of how one could, with the help of appropriate modeling tool, suitably utilize influenza surveillance data to help to provide early signals of an emerging influenza outbreak for the purpose of early detection and swift intervention.
